# Determination of Trace, Micro and Macro Elemental Concentration of Eritrean Honeys

**DOI:** 10.1007/s12011-023-03821-x

**Published:** 2023-08-29

**Authors:** Nikolett Czipa, Béla Kovács, Loránd Alexa, Mehari Gebreyesus

**Affiliations:** 1 Insitute of Food Science, University of Debrecen, Debrecen, Hungary; 2 Department of Agricultural Engineering, Mainefhi, College of Engineering and Technology, May-Nefhi, Eritrea

**Keywords:** Acacia honey, Eritrea, Element content, ICP

## Abstract

In this study macro, micro and trace elemental concentrations were measured in Eritrean acacia honey samples by Inductively Coupled Plasma Optical Emission Spectrometry (Al, B, C, K, Mg, Na, P and S) and Inductively Coupled Plasma Mass Spectrometry (As, Ba, Cd, Co, Cr, Cu, Fe, Mn, Mo, Ni, Pb, Sr and Zn). The concentration of essential elements in the examined Eritrean acacia honeys decreased in the following order: K > P > Ca > Mg > Fe > Zn > Mn > Cu > Sr > Mo. Independent samples T test was used to determine the statistically verified differences between the two regions, but there was none; however there were remarkable differences among the measured element contents of specific honey samples. Elemental concentrations of Eritrean honeys are influenced by the characteristics of the collecting area (e.g. elevation, agricultural activities, water resources).

Our samples showed low essential elemental concentration; therefore the consumption of these honeys does not contribute significantly to the nutrition reference value (NRV) (around 1% of NRV). Toxic elemental concentrations were also low; thus the calculated estimated daily intakes were much lower than the tolerable daily intakes. Consumption of these honeys presents no risk for the human body.

## Introduction

Honey is a natural sweet substance produced by *Apis mellifera* from flower nectar and honeydew. It is a complex food, and its properties depend on environmental and botanical conditions, and postharvest treatments (e.g. storage, filtration, heating) [1]. Honey has low mineral content influenced mainly by soil properties, environmental conditions and floral origin [2]. The element content of soil, water and air of the collecting area also has influence on the element properties of honey [3]. Anthropogenic activities (e.g. burning of fossil fuel, mining, smelting, application of pesticides and fertilizers) may affect soil properties and therefore the element content of honey. Many authors over the world have reported many studies on the importance of the determination of element content of honeys. The most important aims of these researches are: determination of floral and geographical origin of honeys based on elemental concentration, determination of toxic element contents in honeys, and examination of their effects to human body as well as the examination of honeys as environmental indicators. The most commonly used methods for the measurement of elemental concentrations of honeys are ICP-OES (inductively coupled plasma optical emission spectrometry), ICP-MS (inductively coupled plasma mass spectrometry) and AAS (atomic absorption spectrometry).

Based on the data of year 2021, honey production was 151,567 tons in Africa, which is higher than in Oceania (32,924 tons), but much lower than in Asia (859,258 tons), Europe (383,050 tons) or America (345,145 tons). With regard to Africa, honey was produced in the highest quantity in Eastern Africa (74,075 tons) followed by Middle Africa (45,944 tons), Northern Africa (22,699 tons), Western Africa (7765 tons) and Southern Africa (1083 tons). The biggest honey producer in Africa is the United Republic of Tanzania followed by Angola, Kenya, Central African Republic and Ethiopia [4].

Eritrea is an Eastern African country divided into 6 zones, namely South Zone, Maekel Zone, Northern Red Sea Zone, Southern Red Sea Zone, Gash Barka Zone and Anseba Zone. In Eritrea, there are thousands of subsistence and commercial beekeepers. Eritrea has a long history of collecting of honeys from wild or managed bee colonies. The Ministry of Agriculture helps beekeepers with trainings and financial support, therefore the quality and quantity of the produced honey has been improving since 2000. More than 18 000 traditional and more than 17 000 registered bee-colonies are present in Eritrea and there may be thousands of unregistered hives [5]. In Africa, Eritrea is one of the largest honey producing countries because its environmental conditions are excellent for bee farming. In Eritrea, highlands and midlands are ideal for honey production [6]. The Southern region is the most suitable for bee farming, in this region 7677 beekeepers are registered who produce 245 tons of honey annually [7]. Based on the 2021 data of the Ministry of Information Eritrea, honey production increased by 46% compared to 1991 [8]. According to the database of FAOSTAT, [4] the import quantity of natural honey into Eritrea was 3.55 tons in 2019, 3.22 tons in 2020 and 9.31 tons in 2021. The export quantity of Eritrean honey is very low, it was 0.04 tons in 2015 and 0.14 tons in 2019. However, there is no official information on the natural honey production of Eritrea.

In the international literature, the number of articles about the physicochemical parameters of Eritrean honeys is very poor, mainly regarding to the element content of these honeys. Therefore, the main aims of this study were to: (i) determine the trace, micro and macro element content of Eritrean honeys; (ii) compare our results with other research data, (iii) determine the contribution of honey consumption to nutritional reference values; (iv) identify and quantify the toxic elements in our samples and determine the level of risk regarding to these toxic elements.

## Materials and Methods

### Honey Samples

**Fig. 1:**
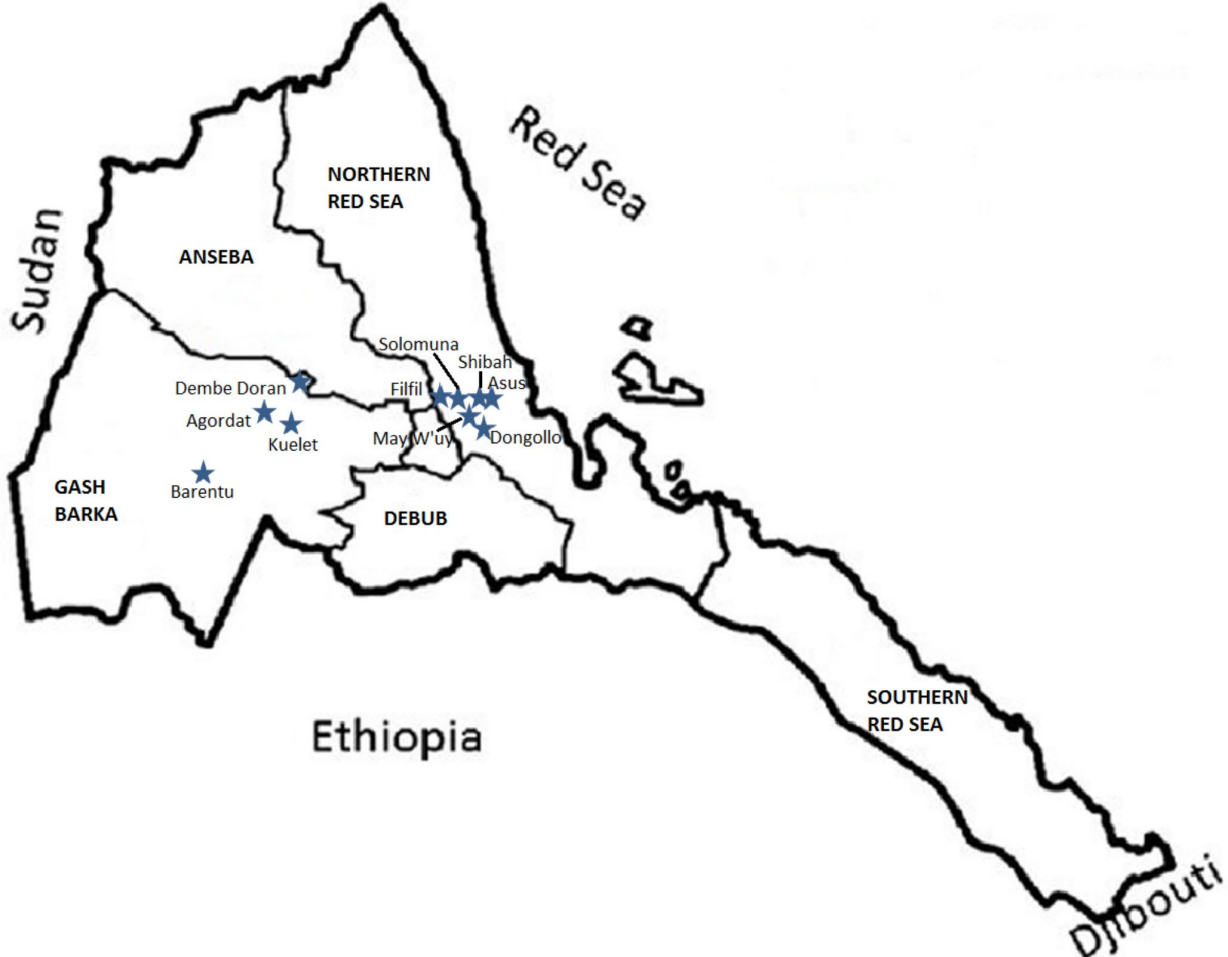
Map of the sample location

Ten acacia honey samples were examined in this study. Samples were collected from Northern Red Sea Zone (samples R1-R6) and Gash-Barka Zone (samples GB1-GB4) directly from local beekeepers in 2020. Each sample was collected from one hive. After centrifugation and filtration, honeys were filled into sterile glass jars; and were stored in the dark at room temperature until the analysis. Locations of the samples: R1 - May W’uy; R2 – Dongolo; R3 – Shibah; R4 – Shebah; R5 – Filfil; R6 - Metkelabet (Asus); GB1 – Dembe-Doran; GB2 – Agordat; GB3 – Barentu; GB4 – Kuelet (Fig. 1). Preparation and examination of the samples were carried out at the University of Debrecen, Hungary in 2021.

### Determination of Element Content

All chemicals were analytical grade. Ultrapure water (18.2 mΩ) was used to prepare the dilutions produced by a Milli-Q water purification system (Millipore S.A.S., Molsheim, France). Digestion of the honey samples was carried out based on the method of Kovács et al. [9]. Three grams of honey was measured into a digestion tube. 10 ml nitric acid (69% v/v; VWR International Ltd., Radnor, USA) was used for the pre-digestion phase (60 °C for 30 min.) and 3 ml hydrogen peroxide (30% v/v; VWR International Ltd., Radnor, USA) was used for the main-digestion phase (120 °C for 90 min.). After the digestion, ultrapure water was added to make a final volume of 50 ml; then the samples were filtered through qualitative filter paper (Sartorius Stedim Biotech S.A., Gottingen, Germany). ICP-OES (Inductively Coupled Plasma Optical Emission Spectrometer) (Thermo Scientific iCAP 6300, Cambridge, UK) was applied for the determination of Al, B and macro elements applying the following wavelengths: 396.153 nm for aluminium, 249.772 nm for boron, 315.887 nm for calcium, 769.896 nm for potassium, 279.806 nm for magnesium, 589.592 nm for sodium, 213.617 nm for phosphorus and 180.7 nm for sulphur. ICP-MS (Inductively Coupled Plasma Mass Spectrometer) (Thermo Scientific XSeries 2, Bremen, Germany) was applied for the determination of other micro elements. The measured isotopes (amu) were the followings: 75 for arsenic, 137 for barium, 111 for cadmium, 59 for cobalt, 52 for chrome, 65 for copper, 56 for iron, 55 for manganese, 95 for molybdenum, 60 for nickel, 206 for lead, 88 for strontium and 66 for zinc.

All analytical determinations were conducted in triplicate.

### Statistical Analysis and Calculations

Data were described by using general terms (mean, standard deviation); Pearson correlation was applied to determine the relation among the examined elements; Independent-Samples T Test was applied to determine the verified differences between the collecting areas; and outliers were determined by using SPSS for Windows, Version 13 (SPSS Inc. Chicago, Illinois, USA).

The daily mineral intake and the contribution to the nutrient reference values (NRVs) was calculated as

$$\text{c}\text{o}\text{n}\text{t}\text{r}\text{i}\text{b}\text{u}\text{t}\text{i}\text{o}\text{n} \left(\text{\%}\right)= \frac{\text{C} \times 100}{\text{N}\text{R}\text{V}},$$

where C is elemental concentration found in 20 g of honey.

The database of Joint FAO/WHO Expert Committee on Food Additives (JECFA) was used for the calculation of risk values. The following equation was used for the calculation:

$$\begin{array}{c}{\rm{Risk}}\, = \,\frac{{{\rm{provisional}}\,{\rm{tolerable}}\,{\rm{daily}}\,{\rm{intake}}\,\left( {{\rm{PTDI}}} \right)\, \times \,{\rm{bodyweight}}\,\left( {{\rm{bw}}} \right)\left( {{\rm{kg}}} \right)}}{{{\rm{concentration}}\, \times \,{\rm{consumption}}}}\\= \,\frac{{{\rm{tolerable}}\,{\rm{daily}}\,{\rm{intake}}\,\left( {{\rm{TDI}}} \right)}}{{{\rm{estimated}}\,{\rm{daily}}\,{\rm{intake}}\,\left( {{\rm{EDI}}} \right)}}\end{array}$$

If the calculated risk value is > 1, there is no adverse health effect are expected, if the calculated risk value < 1, there may be a possible adverse health effect.

## Results and Discussion

Examining the micro element contents of the samples (Table 1), K was present in the highest concentration in all of the examined samples, which aligned with all other studies on honeys. GB3 and GB4 honeys showed the highest K contents, which were much higher than it was measured in the other samples. Similar conclusions can be drawn in case of P and S, which were present in much higher concentrations in these two samples. The highest Ca and Mg contents were determined in sample R3, while sample GB3 showed the highest Na concentration. Sample GB2 showed the lowest concentrations of all the examined elements.

In case of honeys from Northern Red Sea Region, sample R3 (Solomuna) showed the highest Ca, Mg, Na, P and S concentrations. This sample was collected from a rainforest with the elevation between 900 and 2400 m; therefore this was the highest collecting area which has very rich vegetation that can cause these high concentrations. Other samples did not show remarkable differences in their macro element contents. In contrast, honeys of Gash Barka Region showed noticeable differences in their macro elemental concentrations. Sample GB3 (Barentu) and GB4 (Kuelet) showed much higher K, Mg, P and S concentrations than it was determined in the other two samples, and their Ca contents were higher too. Barentu is a lowland, but it was the highest collecting area (1000 m above sea level) of Gash Barka with lots of agricultural areas, which can affect the macro element content of honey. Kuelet is a hill with an elevation of 770 m with forests, which can affect the mineral content of honeys.

**Table 1:** Macro element content (mg/kg), mean ± standard deviation of the examined honey samples

Samples	Ca	K	Mg	Na	P	S
R1	37.5 ± 0.5	169 ± 6	22.8 ± 0.7	25.9 ± 0.4	46.6 ± 0.4	11.5 ± 0.2
R2	45.6 ± 0.6	278 ± 6	17.4 ± 0.2	19.1 ± 0.4	53.4 ± 0.3	22.8 ± 0.5
R3	120 ± 2	180 ± 5	98.5 ± 3.1	31.0 ± 0.6	109 ± 1	24.8 ± 0.5
R4	44.0 ± 0.1	151 ± 2	24.8 ± 0.4	23.5 ± 0.6	47.6 ± 0.4	14.4 ± 0.2
R5	46.9 ± 0.5	161 ± 2	17.7 ± 0.5	15.2 ± 0.1	42.8 ± 0.3	20.8 ± 0.1
R6	36.4 ± 0.8	205 ± 2	21.3 ± 0.1	23.7 ± 0.3	54.6 ± 0.9	21.5 ± 0.5
GB1	27.3 ± 0.2	113 ± 2	9.64 ± 0.06	15.9 ± 0.2	43.1 ± 0.4	17.0 ± 0.7
GB2	12.9 ± 0.3	86.7 ± 0.8	6.30 ± 0.05	5.10 ± 0.2	32.0 ± 0.4	10.7 ± 0.3
GB3	57.6 ± 0.2	1560 ± 2	90.3 ± 0.4	44.7 ± 0.2	294 ± 1	169 ± 3
GB4	38.2 ± 0.6	1229 ± 33	79.2 ± 2.0	15.8 ± 0.4	202 ± 2	120 ± 2

Based on the results of Pearson correlation analysis, very strong relations (**) were determined between K and P (0.969), K and S (0.996), Mg and P (0.826), P and S (0.981) at the level of 0.01. There were moderate correlations (*) between Ca and Mg (0.729), K and Mg (0.694), Mg and Na (0.663), Mg and S (0.715), Na and P (0.655) at the level of 0.05.

According to the results of T test, there were no statistically verified difference between the samples of Eastern Red Sea and Gash-Barka regions. P values were the followings: 0.275 for Ca, 0.239 for K, 0.613 for Mg, 0.721 for Na, 0.280 for P and 0.231 for S.

Examining the micro elemental concentrations (Table 2), B was measured in the highest concentration in all of the examined samples. Al was the second most abundant element in case of sample R1, R4, R5 and GB1; while Fe was present in higher concentration than Al in the other honeys. The highest Al content was observed in sample GB1, sample R5 showed the highest B concentration, and the highest Sr content was measured in sample R3. Sample GB3 showed the highest Fe and Zn contents; the highest Ba and Mn concentrations were measured in sample R2; while Cu was determined in the highest concentration in sample GB4. Cu and Mn contents were much higher (more than 10-fold for Cu) in sample GB3 and GB4 than in the other samples. Ba showed the lowest contents except for sample R1 and R2, in which Cu was determined in the lowest concentration. Sample GB2 showed the lowest Al, B, Fe, Mn and As concentrations, the lowest Cu and Zn contents were determined in sample R1, while sample GB4 showed the lowest Ba concentration.

**Table 2:** Micro element content (µg/kg), mean ± standard deviation of the examined honey samples

Sample	Al	B	Ba	Cu	Fe	Mn	Sr	Zn
R1	1057 ± 58	4847 ± 64	44.9 ± 2.1	32.0 ± 0.4	904 ± 13	406 ± 1	156 ± 2	287 ± 6
R2	1348 ± 14	3510 ± 41	103 ± 2	42.1 ± 0.5	1450 ± 14	1185 ± 6	188 ± 1	387 ± 5
R3	1765 ± 13	5719 ± 82	74.0 ± 0.9	80.7 ± 1.7	1773 ± 20	801 ± 5	406 ± 9	481 ± 1
R4	1501 ± 78	4573 ± 33	45.3 ± 0.3	369 ± 2	1404 ± 7	414 ± 3	148 ± 2	574 ± 6
R5	1132 ± 17	6156 ± 52	33.7 ± 0.2	103 ± 3	1113 ± 6	528 ± 2	205 ± 5	602 ± 1
R6	1322 ± 27	4010 ± 20	33.3 ± 0.2	79.8 ± 1.5	1566 ± 26	327 ± 3	170 ± 3	368 ± 5
GB1	2076 ± 52	3602 ± 73	35.8 ± 0.4	52.4 ± 1.3	1932 ± 19	251 ± 1	141 ± 2	1698 ± 9
GB2	413 ± 5	2814 ± 13	33.3 ± 0.8	34.3 ± 0.3	495 ± 6	129 ± 3	60.1 ± 0.5	332 ± 1
GB3	1606 ± 47	5080 ± 87	34.1 ± 0.1	545 ± 4	3346 ± 6	662 ± 4	299 ± 3	2394 ± 7
GB4	1284 ± 31	3116 ± 38	31.7 ± 0.3	605 ± 3	2329 ± 5	601 ± 5	199 ± 2	1717 ± 14

In case of honeys from Northern Red Sea Region, the acacia honey collected in the rainforest (R3) showed the highest Al, Fe and Sr concentrations, sample R2 (Dongollo) showed the highest Ba and Mn contents, and the highest B and Zn concentrations were determined in sample R5 (Filfil mountain). Examining the samples from Gash Barka, GB3 and GB4 honeys showed the highest Cu, Fe, Mn, Sr and Zn concentrations, the highest Al content was determined in sample GB1, while sample GB3 showed the highest B concentration.

Very strong (**) correlation was determined between Ba and Mn (0.826), Fe and Zn (0.877) at the level of 0.01; and moderate correlation (*) was observed between B and Sr (0.640), Cu and Fe (0.729), Cu and Zn (0.717) at the level of 0.05.

According to the results of T test, there were no statistically verified difference between the samples of Eastern Red Sea and Gash-Barka regions. P values were the followings: 0.976 for Al, 0.114 for B, 0.108 for Ba, 0.309 for Cu, 0.220 for Fe, 0.339 for Mn, 0.573 for Sr and 0.086 for Zn.

Examining the trace elemental concentrations, Mo was the only examined trace element that was measureable in every honey sample; the highest concentration was determined in sample GB3, which was multiple of the Mo content of other honeys. Arsenic contents were under the limit of detection (LoD = 2.10 µg/kg) in sample R5 and R6; while the highest arsenic concentration was detected in sample GB4. The other samples showed similar arsenic contents. Similarly to Mo, sample GB3 showed the highest Ni concentration followed by sample R4 and R5 (similar concentrations), and sample R1. Ni content was under the limit of detection (LoD = 9.61 µg/kg) in the other honeys. Co content was above LoD (13.1 µg/kg) only in three samples (R1, R3 and R5); while Cd and Cr contents were in detectable concentration only in sample R1 (it is collected from a hot spring zone); other honeys contained these elements under LoD (10.1 µg/kg for Cd and 15.0 µg/kg for Cr). The concentration of Pb was above LoD (7.50 µg/kg) only in five samples (R1, R2, R4, R5 and BG4) with similar values (Table 3).

In case of samples from Red Sea Region, R1 honey showed the highest trace elemental concentrations (except for Ni). The collecting area of this sample was the May W’uy area, where hot springs can be found. Similarly high As, Mo, Pb and higher Ni concentrations were measured in sample R2 collected from Dongollo, which is famous of its radioactive waters. Examining samples from Gash Barka Region, GB3 and GB4 honeys showed higher As, Mo and Ni concentrations than the other two samples.

Very strong (**) correlation was determined between As and Co (1.00), Ni and Co (1.00), Pb and Co (1.00) at the level of 0.01.

**Table 3:** Trace element content (µg/kg), mean ± standard deviation of the examined honey samples

Sample	As (µg/kg)	Cd (µg/kg)	Co (µg/kg)	Cr (µg/kg)	Mo (µg/kg)	Ni (µg/kg)	Pb (µg/kg)
R1	3.69 ± 0.05	15.9 ± 0.1	25.9 ± 0.3	15.4 ± 0.1	12.3 ± 0.7	16.2 ± 0.1	14.2 ± 0.2
R2	3.67 ± 0.07	< 10.1	< 13.1	< 15.0	14.1 ± 0.3	36.2 ± 0.2	14.7 ± 0.1
R3	2.82 ± 0.05	< 10.1	21.6 ± 0.5	< 15.0	4.57 ± 0.05	< 9.61	< 7.50
R4	2.21 ± 0.03	< 10.1	< 13.1	< 15.0	3.60 ± 0.04	28.5 ± 0.2	12.8 ± 0.5
R5	< 2.10	< 10.1	28.4 ± 0.2	< 15.0	2.91 ± 0.02	29.2 ± 0.4	11.5 ± 0.2
R6	< 2.10	< 10.1	< 13.1	< 15.0	4.65 ± 0.04	< 9.61	< 7.50
GB1	2.17 ± 0.04	< 10.1	< 13.1	< 15.0	3.28 ± 0.02	< 9.61	< 7.50
GB2	3.03 ± 0.05	< 10.1	< 13.1	< 15.0	4.35 ± 0.08	< 9.61	< 7.50
GB3	3.62 ± 0.01	< 10.1	< 13.1	< 15.0	113 ± 1	53.1 ± 0.1	< 7.50
GB4	5.41 ± 0.07	< 10.1	< 13.1	< 15.0	22.2 ± 0.4	18.2 ± 0.2	13.7 ± 0.2

According to the results of T test, there were no statistically verified difference between the samples of Eastern Red Sea and Gash-Barka regions. P values were the followings: 0.574 for As, 0.353 for Mo, 0.723 for Ni (in case of other elements T test was not applicable).

Examining the macro, micro and trace elemental concentrations, more strong (**) and moderate (**) correlations were found. Strong correlations were between the following elements: Ca and Sr (0.944), Ca and Ni (0.975), K and Cu (0.868), K and Fe (0.846), K and Zn (0.815), K and Co (-0.998),.K and Mo (0.855), Mg and Sr (0.847), P and Cu (0.823), P and Fe (0.890), P and Zn (0.802), P and Mo (0.876), S and Cu (0.856), S and Fe (0.865), S and Zn (0.831), S and Mo (0.879), Fe and Mo (0.791) at the level of 0.01. Moderate correlations were between the following elements: Ca and B (0.646), Mg and Fe (0.720), Na and Fe (0.703), Na and Sr (0.715), Na and Mo (0.730), Zn and Mo (0.734) at the level of 0.05.

There is no scientific report on the element content of Eritrean acacia honeys, therefore our results were compared to studies representing other countries (Table 4). These acacia honeys were collected in Saudi Arabia, Jordan, Malaysia, Hungary, Croatia, Serbia, Slovakia, Poland and Italy. In this analysis, outliers were not involved.

Examining the macro element contents, K was the most abundant elements in all honeys from different countries. Saudi Arabian and Malaysian honeys showed the highest K contents followed by Slovakian and Croatian samples. The lowest concentrations were reported in Serbian acacia honeys; the other samples showed similar K contents to our honeys’. Saudi Arabian and Croatian samples showed higher Ca concentrations than it was observed in honeys from other countries. Saudi Arabian samples showed much higher Mg concentrations than other honeys; and the concentration of this element was higher in our samples too. In case of Na, there were no remarkable difference among acacia honeys from different countries, but the highest contents were observed in Malaysian samples. The highest P contents were determined in the current study. S contents were measured only in our and Hungarian acacia honeys, these concentrations were similar.

Jordanian, Serbian and Slovakian acacia honeys showed higher Al concentrations than it was determined in this study or in Italian and Croatian samples. Similar Cu contents were determined in our samples and in Malaysian and Croatian honeys. Extremely high Fe contents were reported in Saudi Arabian honeys, but the concentration of this element was very high in Jordanian and Malaysian samples too. Similar Fe contents were determined in our and Polish honeys, which were lower than in Serbian honeys. Saudi Arabian acacia honeys showed very high Mn concentrations, which were multiple of Mn contents measured in any acacia honeys from other countries. In our samples, the concentration of this element showed wide variation like Polish and Italian honeys. Most of the acacia honeys presented a wide range of Zn content (except for Slovakian samples); Saudi Arabian honeys showed higher concentrations, however the highest Zn contents were measured in Polish honeys. B, Ba and Sr contents were published only by a few research; examining the higher concentrations, our samples showed higher B and Sr contents than Hungarian and Jordanian acacia honeys; while Croatian and Jordanian samples showed higher Ba contents than it was measured in our samples.

Examining trace elements, arsenic was measured in our, Jordanian, Croatian and Italian honeys in similar concentrations. In case of Cd, very high concentrations were determined in Saudi Arabian honeys, which were at least one order of magnitude higher than in other acacia honeys. Jordanian and Saudi Arabian honeys compared to other samples showed extremely high Co concentrations, which were much higher than in the current study or other studies involved. Jordanian, Slovakian and Italian acacia honeys showed higher Cr concentrations than in the current study. There were no noticeable difference among Mo concentrations of acacia honeys from Eritrea, Jordan, Croatia and Italy. Similar Ni concentrations were measured in Jordanian and Slovakian acacia honeys and the current study; however Saudi Arabian, Croatian and Italian honeys showed higher Ni contents. In case of Pb content, Saudi Arabian honeys showed high concentrations, other samples showed lower Pb contents.

**Table 4:** Comparison among the element contents of Eritrean acacia honeys and acacia honeys from other countries

Elements	Our samples	Saudi Arabian [10]	Jordanian [11]	Malaysian [12]	Hungarian honeys [3]	Croatian honeys [13]	Serbian [14]	Slovakian honeys [15]	Polish honeys [16]	Italian honeys [17]
Ca (mg/kg)	12.9–57.6*	96.7–99.9	54 ± 16	40.6 ± 1.64	nd	78–140	nd	20.3 ± 3.09	28.6–69.2	nd
K (mg/kg)	86.7–278*	429–491	222 ± 38	467 ± 5.17	132–237	235–960	46.4–63.5	340 ± 27	127–196	nd
Mg (mg/kg)	6.30–98.5	189–196	14.5 ± 1.6	6.34 ± 0.09	2.48–10.2	4.3–46	5.71-12.0	12.5 ± 2.44	6.50–14.0	nd
Na (mg/kg)	5.10–31.0*	15.9–19.0	20 ± 1.5	42.7 ± 1.88	1.30–6.40	5.1–25	6.75–12.5	8.49 ± 1.10	3.80–42.8	nd
P (mg/kg)	42.8–202*	29.0-30.3	42 ± 3.5	nd	21.7–76.7	nd	nd	nd	7.14–28.6	nd
S (mg/kg)	11.5–24.8*	nd	nd	nd	6.40–22.7	nd	nd	nd	nd	nd
Al (µg/kg)	413–2076	nd	2710 ± 190	nd	nd	360–550	2090–2600	4310 ± 380	nd	520 ± 350
B (µg/kg)	3116–6156	nd	nd	nd	2040–4140	nd	nd	nd	nd	nd
Ba (µg/kg)	31.7–45.3*	nd	80 ± 4	nd	nd	11–111	nd	nd	nd	nd
Cu (µg/kg)	32.0-605	nd	196 ± 34	410 ± 210	nd	66–490	nd	83.8 ± 13.5	100–200	300 ± 180
Fe (µg/kg)	495–2329*	92,560–98,130	12,600 ± 6040	12,800 ± 4690	25–584	310–1100	3640–3910	1020 ± 210	400–2900	1300 ± 530
Mn (µg/kg)	129–1185	5250–6040	201 ± 72	nd	nd	110–420	540–660	440 ± 53	100–700	610 ± 376
Sr (µg/kg)	60.1–406	nd	80 ± 4	nd	nd	nd	nd	nd	nd	nd
Zn (µg/kg)	287–2394	4360–5720	850 ± 414	1170 ± 300	74-3370	nd	380–1080	159 ± 13	200–9500	910 ± 230
As (µg/kg)	< 2.10–5.41	nd	1.5 ± 1.6	nd	nd	< 2	nd	nd	nd	7.7 ± 17
Cd (µg/kg)	< 10.1–15.9	110–160	2.3 ± 1.7	20 ± 0	<LoD-0.571	< 1	nd	1.17 ± 0.18	<LoD	nd
Co (µg/kg)	< 13.1–21.6	1150–1320	4000 ± 1700	nd	nd	51 ± 31	nd	nd	< 100–200	8.5 ± 0.5
Cr (µg/kg)	< 15.0-15.4	nd	160 ± 47	nd	<LoD	24 ± 9.7	nd	26.8 ± 3.59	<LoD	86 ± 68
Mo (µg/kg)	2.91–22.2*	nd	23 ± 3	nd	nd	20 ± 10	nd	nd	nd	4.1 ± 20
Ni (µg/kg)	< 9.61–53.1	510–660	53 ± 13	nd	nd	281 ± 260	nd	23.4 ± 3.8	< 200–300	130 ± 77
Pb (µg/kg)	< 7.50–14.7	190–230	20 ± 5.8	nd	nd	< 2–14	nd	9.40 ± 1.13	<LoD	71 ± 213

*Outliers: Ca: 120 mg/kg (R3); K: 1229 mg/kg (GB4) and 1560 mg/kg (GB3); Na: 44.7 mg/kg (GB3); P: 294 mg/kg (GB3); S: 120 mg/kg (GB4) and 169 mg/kg (GB3); Ba: 74.0 µg/kg (R3) and 103 µg/kg (R2); Fe: 3346 µg/kg (GB3); Mn: 1185 µg/kg (R2); Mo: 113 µg/kg (GB3); Sr: 406 µg/kg (R3) and 299 µg/kg (GB3);

nd = not determinated

### Evaluation of the Effect of the Elemental Composition of Samples on Human Health

In many research, it was reported that the contribution of honey consumption to NRVs is very low. In this study, Regulation (EU) No. 1169/2011 [18] was considered that involves the reference intakes for minerals. In the calculation, the highest measured concentrations were analysed assuming a daily consumption of 20 g (one tablespoon) of honey. Table 5 shows the contributions to NRVs examining our acacia honeys. The highest contribution was 4.52% in case of Mo and the lowest (0.30%) in case of Ca.

**Table 5:** Contributions to NRVs

Elements	NRVs	Content in 1000 g of honeys	Content in 20 g of honey	Contribution to NRV
Ca	800 mg	120 mg (R3)	2.40 mg	0.30%
K	2000 mg	1560 mg (GB3)	31.2 mg	1.56%
Mg	375 mg	98.5 mg (R3)	1.97 mg	0.53%
P	700 mg	294 mg (GB3)	5.88 mg	0.84%
Cu	1000 µg	605 µg (GB4)	12.1 µg	1.21%
Fe	14,000 µg	3346 µg (GB3)	66.9 µg	0.48%
Mn	2000 µg	1185 µg (R2)	23.7 µg	1.19%
Zn	10,000 µg	2394 µg (GB3)	47.9 µg	0.48%
Cr	40 µg	15.4 µg (R1)	0.308 µg	0.77%
Mo	50 µg	113 µg (GB3)	2.26 µg	4.52%

Honeys may contain toxic elements (e.g. As, Pb, Cd), which have effects on human health. Joint FAO/WHO Expert Committee on Food Additives (JECFA) determined the tolerable intakes for different metals, which have toxic effect on human health. Table 6 contains the PTDIs and the results of the risk assessment.

Based on these results, the examined Eritrean honeys have no toxic effect on human health by the above-mentioned consumption volume. The lowest risk value was calculated in case of Cd content of sample R1; from which a child with 30 kg bw (body weight) should consume 1.56 kg per day to reach the risk value of 1.

**Table 6:** Results of the risk assessment for Eritrean honeys

Metals	PTDI (µg/kg bw)	References	Risk value for 30 kg bw	Risk value for 60 kg bw	Risk value for 90 kg bw
Al	286	[19]	206	413	619
As	withdrawn (2.14)	[20]	593	1187	1780
Cd	0.833	[21]	79	157	236
Cu	500	[22]	1240	2479	3719
Fe	800	[23]	359	717	1076
Pb	withdrawn (3.57)	[24]	364	729	1093
Zn	300–1000	[22]	188	376	564

## Conclusions

The concentration of essential elements in the examined Eritrean honey samples decreased in the following order: K > P > Ca > Mg > Fe > Zn > Mn > Cu > Sr > Mo.

In case of acacia honeys from lowlands (R4, R6, GB1, GB2 and GB3), sample GB2 (Agordat – 600 m) showed the lowest macro and micro elemental concentrations, and sample GB3 (Barentu – 1000 m) showed the highest micro and macro element contents (except for Al, where GB1 showed the highest concentration). These samples were collected from lowlands with different elevation, therefore it can be stated that sample from the highest lowland (GB3) showed the highest concentrations. It should be noted that in case of the other 4 samples, the honeys from higher lowland showed lower concentrations in case of many elements. Therefore we can state that climate can also affect the element content of honeys, and since the agriculture is very common in these areas, it might have an effect to the micro and macro element content of honeys. In case of Ca, K, Mg, Na, B, Cu and Mn contents, the order was the following: GB2 < GB1 < R6 < R4 < GB3.

In case of the samples from highlands, R3 honey (Solomuna – above 1000 m) showed the highest Ca, Mg, Na, Al and Sr contents, sample R5 (Filfil – 800 m) showed the highest B content, sample R2 (Dongollo – 900 m) showed the highest Ba and Mn concentrations and sample GB4 (Kuelet hill – 700 m) showed the highest K, P, S, Cu, Fe and Zn contents. Therefore the conclusion in case of these honeys cannot clear, so we can state that the element content of these honeys are influenced by e.g. geographical area, soil properties, climate and vegetation.

Because the concentrations of the examined essential elements were very low; the contribution of honey consumption to NRV is negligible (around 1%). Eritrean acacia honeys contain toxic elements (e.g. As, Cd, Pb); however the estimated daily intake of these elements in the examined honeys indicated that the estimated intakes were within the WHO tolerable daily intakes. Therefore the consumption of these honeys does not cause any problem in the human body.

Because there is no scientific report available on the elemental concentration of Eritrean honeys, this manuscript can be a good starting point for further research. Collection of honeys from more zones of Eritrea and the analysis of honey samples in larger number can help Eritrean researchers to get into the scientific life.

## Data Availability

The datasets used and analysed during the current study are available from the corresponding author on a reasonable request.
